# Translation and Validation of a State-Measure of Body Image Satisfaction: The Body Image State Scale

**DOI:** 10.3389/fpsyg.2021.724710

**Published:** 2021-10-28

**Authors:** Luc Bardi, Claire Arnaud, Céline Bagès, Frédéric Langlois, Amelie Rousseau

**Affiliations:** ^1^Université de Lille, ULR 4072 – PSITEC – Psychologie: Interactions, Temps, Emotions, Cognition, Lille, France; ^2^Laboratoire sur l’anxiété et le Perfectionnisme, Department of Psychology, Université du Québec à Trois-Rivières, Trois-Rivières, QC, Canada; ^3^Centre d’Etudes et Recherches en Psychopathologie et Psychologie de la Santé (CERPPS), Université de Toulouse (UT2J), Toulouse, France

**Keywords:** body satisfaction, state measure, validation, French, body image

## Abstract

The aim of the present study is to test the validity and reliability of the French Body Image State Scale (F-BISS). The scale was translated using a back-translation technique, with discrepancies being settled through consensus. Three hundred and twelve female participants were recruited. Convergent validity was assessed using eating disorder evaluation and social comparison. Exploratory and confirmatory factor analyses were also conducted. The translated Body Image State Scale (BISS) demonstrated good psychometric properties, with good internal consistency (α = 0.83), and adequate goodness-of-fit. The translated BISS presented a unifactorial structure, with one factor explaining 56% of the variance. The exploratory factor analysis led to the removal of a single item due to insufficient factor loading (<0.45). Its convergent validity seems consistent with previous literature. Discriminant analyses showed a significant difference in F-BISS score between participants relative to eating disorder symptomatology (*t* = 11.65; *p* < 0.001). This translation could prove useful in both research and clinical settings to assess state body satisfaction in French populations.

## Introduction

A growing body of research warns of the public health issue represented by body dissatisfaction, or negative body assessment (Bucchianeri and Neumark-Sztainer, 2014; Griffiths et al., 2017; Bornioli et al., 2019). Developing reliable measurement tools or validating existing ones in multiple languages in this context allows researchers to better understand the onset of eating disorder development. Indeed, body dissatisfaction is a risk factor for eating disorders (Stice, 2002; Stice et al., 2011). In western countries, women are pressured to achieve a thin body-ideal and western sociocultural influences have been proven to be a risk factor for general population body dissatisfaction (Holmqvist and Frisén, 2010). In France, men’s body dissatisfaction differs from women’s, with men being more preoccupied about their muscularity and gaining weight (Pope et al., 2000) and women being more concerned about being thin and toned (Girard et al., 2018). In this study, we will focus on state-measurement tools, as they have raised criticism in the way some were developed and validated in the past (Cash et al., 2002; Bateson et al., 2007). More precisely, we will focus on the validation of a positive body assessment, or body satisfaction, state scale: the Body Image State Scale (BISS) (Cash et al., 2002).

Body dissatisfaction or satisfaction are indeed variably conceptualized in two ways, “state,” and “trait,” meaning it can be viewed as an immediate state of being, or a stable personality trait (Cash et al., 2002). Multiple trait body-dissatisfaction scales have been translated in French, such as the Body Shape Questionnaire (BSQ; Cooper et al., 1987; Rousseau et al., 2005), the body-dissatisfaction subscale of the EDI (Garner et al., 1983; Archinard et al., 1996), the Eating Disorder Examination Questionnaire’s (EDE-Q’s) shape and weight concern subscales (Fairburn and Beglin, 1994; Fairburn, 2008; Carrard et al., 2015), or the Male Body Dissatisfaction Scale (Ochner et al., 2009; Rousseau et al., 2014). The use of trait-scales lie in evaluating body-dissatisfaction in a punctual fashion, for research or clinical purposes such as determining if a patient is suitable for therapy. Moreover, trait-measures are often retrospective, and do not seem much suited for assessing change over a shorter period of time. Therefore, life-changes that may harm body image such as receiving a surgery (Sarwer et al., 2010) could only be assessed after a period of time when using a trait-measure. Since negative body image is linked to pathology, having to wait to correctly assess it could hinder patients’ treatment. Cash et al. (2002) emphasized the fact that most measures of body dissatisfaction or satisfaction are trait measures, such as the BSQ (Cooper et al., 1987). To the research team’s knowledge this statement holds true today in France with only one figure or contour-drawing based state scale being validated in French (Moussally et al., 2017) and another Contour-Drawing Rating Scale (CDRS; Thompson and Gray, 1995) being used in some research with French-speaking samples (Duchesne et al., 2017; Rivière et al., 2018) with seemingly no prior validation. However, this type of scale has been criticized for not being representative enough of female body diversity (Bateson et al., 2007). When searching for the keywords “body-dissatisfaction” and “state scale” and “French,” no relevant results are found on the PsycNet or ScienceDirect databases.

Cash et al. (2002) also underlined the issue that tools used to measure state body satisfaction in previous research have often been developed from trait-scales, or constructed without prior validation. As said earlier, these scales may lack sensitivity. For this purpose, Cash et al. (2002) developed the Body-Image State Scale. This 6-item state-measure of body satisfaction is non-specific, exclusively text-based and originally available in English. Its 9-point scale with specific body-related statements to choose from should allow participants to give a precise answer. It is less prone to the unrealistic body representation associated with contour-drawing based scales, as participants are asked about their own body, without being shown one. The BISS has been widely used in previous literature (i.e., Etu and Gray, 2010; Walker et al., 2012; Boersma and Jarry, 2013). Currently, Spanish, Italian, and Dutch versions have been developed, with only the Spanish version being the subject of a validation study (Carraro et al., 2010; Alleva et al., 2014; Mebarak Chams et al., 2019). Convergent validity has been tested using the BSQ (Cooper et al., 1987), and the EDE-Q (Fairburn and Beglin, 1994). Validation studies have shown very good psychometric qualities (Cash et al., 2002; Mebarak Chams et al., 2019). The BISS appears to have a single-factor structure, with every item loading on the main factor at a >0.75 coefficient (Mebarak Chams et al., 2019). Convergent validity across studies also appears coherent with literature: the BISS had a negative correlation with body-dissatisfaction (*r* = −0.52, *p* < 0.001; Cash et al., 2002; *r* = −0.58, *p* < 0.001; Mebarak Chams et al., 2019), eating disorder symptomatology (*r* = −0.79, *p* < 0.01; Alleva et al., 2014) and general psychopathology (*r* = −0.33, *p* < 0.001; Mebarak Chams et al., 2019). Discriminant analyses also showed a significant difference between Body Mass Index (BMI) groups with participants having a higher BMI being less satisfied than those with a lower BMI (Mebarak Chams et al., 2019). However, no study has yet replicated a long-term test–retest procedure like in the original study. Indeed, the only test–retest data available comes from Carraro et al. (2010), who assessed test–retest fidelity only an hour after an experimental task (*r* = 0.87, *p* < 0.01). The BISS appears to have been neither translated nor validated into French.

Validation of the BISS in French would be of use for a variety of professions. State scales as said are indeed very useful in research and clinical settings. In a research setting, they may be used to assess the immediate impact of an exposure task such as the ones used in the study of social media’s impact on body image (Tiggemann and Zaccardo, 2015; Cohen et al., 2019). In a clinical setting, they could be used by the therapist as a self-evaluation tool for patients to better understand what causes body image-related distress, and assess differences over time. The BISS is even more relevant for patients’ use, as it is a short and easy scale to use.

To validate the BISS, it seemed important to take notice of the differences in body dissatisfaction expression between men and women’s body image. Indeed, research has shown men tend to express dissatisfaction related to their muscle mass (Karazsia et al., 2017). As mentioned earlier, this finding seems to hold true in a French sample, with men wanting to gain weight and muscle mass (Pope et al., 2000). French women, on the other hand, seem to be pressured to reach a more thin and toned body-shape (Girard et al., 2018). Moreover, 70% of French women want to lose weight (Valls et al., 2013) and 34.6% state that their self-opinion depends on their weight (Lachaud et al., 2004). While those results underline the importance of validating a state-measure in French to adequately assess immediate body-satisfaction, this would also mean we would need different measures of convergent validity for each gender. Therefore, we wished to focus at first on a female population.

Secondly, elements in research made us consider validating the BISS in a younger population. Indeed, research showed that cut-off scores to the EDE-Q (Fairburn and Beglin, 1994; Fairburn, 2008) varied between age groups, being higher in a younger population (Rø et al., 2015). Moreover, older women tend to display a lesser desire, or drive, to be thinner (Pruis and Janowsky, 2010); this would be a bias since tools available for this study were mainly validated in a younger population and addressed thinness-related preoccupations.

The main objective of this study is therefore to translate and validate the BISS (Cash et al., 2002) in an 18–25-year-old female French population.

It was expected here that a French version of the BISS would negatively correlate with measures of body dissatisfaction, social comparison, and eating symptomatology, and would present a single-factor structure, as demonstrated in relevant research (Mebarak Chams et al., 2019). Moreover, we wished to replicate differences between BMI groups and eating disorder symptomatology groups found in the Mebarak Chams (2019) study; it is expected that higher BMI categories will have a lesser French Body Image State Scale (F-BISS) score than lower BMI groups. It is also expected that participants with a clinically significant score to an eating disorder symptomatology scale will have a lower score than participants with a clinically non-significant score (Mebarak Chams et al., 2019).

## Materials and Methods

### Participants

The study included 312 participants. Seventy-eight participants completed the test two times at a 2-week interval (*N*_Time__1_ = 312; *N*_Time__2_ = 78). Participants were 18–25-year-old female students (*M* = 21.07, SD = 1.82). Self-reported values of height and weight were used to derive BMI (*M* = 22.88, SD = 4.48). Participants did not receive compensation. For sample size, Everitt’s (1975) recommendations of a subject to item ratio of 10 were used.

### Procedure

This study received approval by the University’s Board of Ethics. Recommendations for translation from Cha et al. (2007) were followed. The scale was translated into French by several members of the research team, one of whom was fluent in English (C1 level; Council of Europe, n.d.). Due to a lack of independent translators, the translation was then back-translated by another English-fluent team member and reviewed by the research team. Minor discrepancies were settled through consensus. An advertisement for a study on body image was posted on various French-speaking student Facebook groups, with a message stating the research team’s intent to recruit 18–25-year-old female participants. Willing participants had to fill out the Body Shape Questionnaire 8-item (BSQ-8C), the EDE-Q, the Physical Appearance Comparison Scale-4 (PACS-4), the BISS, and a sociodemographic data questionnaire, in that order. When ending the questionnaire, participants were asked to create a code (enabling anonymous test–retest analyses) consisting of the two last digits of participants’ phone number, the first two letters of their first name and the last two digits of their birth year. Participants were then asked if they wished to take part in the study’s second phase. If they did, they had to input their participant code again and provide their email address. Willing participants received an email 2 weeks later with a link to another questionnaire. To replicate the original study, we chose to respect the same 2-week delay between questionnaire answering (Cash et al., 2002). A 2-week interval also seems to be considered the highest appropriate interval for retest of a state-scale (Polit, 2014). The questionnaire contained the French BISS, as well as another 4-item questionnaire to be validated in another study. Participants were again prompted to input their code. Finally, a written debrief was sent *via* email.

### Study Material

#### Body Image State Scale

This 6-item self-reported scale (Cash et al., 2002) is rated on a 9-point Likert scale. Each item begins with the sentence “Right now, I feel” [for instance: “Right now, I feel (Extremely dissatisfied to Extremely satisfied) with my physical appearance”]. Every rating’s phrasing is different. For instance, item 1 ranges from Extremely dissatisfied (1) to Extremely satisfied (9), while item 4 ranges from Extremely physically attractive (9) to Extremely physically unattractive (1). Score is a mean of every item. Higher scores denote higher body satisfaction. Half items have reverse rating (2, 4, 6). Internal consistency calculated by Cronbach’s alpha in the original study is 0.77 in women, and 0.83 in this study’s sample.

#### Body Shape Questionnaire 8-Item

This self-reported questionnaire was developed by Cooper et al. (1987), and abbreviated by Evans and Dolan (1993). This abridged version has been validated in French by Lentillon-Kaestner et al. (2014). This 8-item questionnaire measures body dissatisfaction over the past 4 weeks [item example: “Has thinking about your shape interfered with your ability to concentrate (e.g., while watching television, reading, listening to conversations?”)]. A total score of trait body dissatisfaction is obtained by adding the score for each Likert-scale item (1–6; maximum score of 48). A higher score denotes higher body dissatisfaction. In this study’s sample, internal consistency measured by Cronbach’s alpha is 0.93, and scores range from 8 to 48, with a mean score of 25.35 (SD = 10.47).

#### Eating Disorder Examination Questionnaire

This self-reported questionnaire (Fairburn and Beglin, 1994; Fairburn, 2008), validated in French by Carrard et al. (2015), assesses eating disorder symptomatology. Items include questions such as “Have you gone for long periods of time (8 waking hours or more) without eating anything at all in order to influence your shape or weight?” Answers are on a 7-point Likert scale. While the questionnaire was originally designed with four subscales in mind, it is advised to only use the overall score, as a single-factor structure is thought to be more robust (Friborg et al., 2013). The four subscales are restraint (items 1, 2, 3, 4, 5; α = 0.84), eating concerns (items 7, 9, 19, 21, 20; α = 0.80), shape concerns (items 6, 8, 23, 10, 26, 27, 28, 11; α = 0.91), and weight concerns (items 22, 24, 8, 25, 12; α = 0.89). Cut-off scores from Rø et al. (2015) study were used. While Rø et al. (2015) evaluated Norwegian adults, and warned about eventual cross-cultural differences, a lack of similar data in the French validation article (Carrard et al., 2015) forced us to use a near cultural equivalent for the same scale. For an underweight BMI, cut-off was 1.62. For a normal BMI, cut-off was 2.51. For an overweight BMI, cut-off was 3.15. Finally, for an obese BMI, cut-off was 3.26. A score superior to these values was considered clinically significant. In this study’s sample, internal consistency measured by Cronbach’s alpha is 0.91, and scores range from 0 to 5.57, with a mean score of 1.95 (SD = 1.40).

#### The Physical Appearance Comparison Scale-4

This self-reported 4-item questionnaire (Thompson et al., 1991; Dany and Urdapilleta, 2012) measures the general tendency of individuals to compare themselves with others in social situations (item example: “At parties or other social events, I compare my physical appearance to the physical appearance of others.”). Participants are presented a Likert scale ranging from 1 (never) to 5 (always). Overall score is calculated by adding each item’s individual score. The higher the score (maximum score of 20), the more likely the individual is to use social comparison. In this study’s sample, internal consistency measured by Cronbach’s alpha is 0.84, and scores range from 4 to 20, with a mean score of 11.92 (SD = 3.85).

#### Sociodemographic Data Questionnaire

This questionnaire was used to report on participants’ age, education level, and area of study. As some parts of this questionnaire were explicitly meant for female participants (EDEQ items on menstruation and contraceptive pill usage), and since the recruitment campaign was explicitly directed at cisgendered women 18–25 years old, no questions about gender were added.

### Statistical Analyses

Construct validity was assessed using exploratory and confirmatory factor analyses, as well as inter-item correlations. The exploratory factor analysis was conducted on SPSS 25, while the confirmatory factor analysis was carried out on RStudio using the lavaan package (Rosseel, 2012). Both used a maximum likelihood estimation method. For the factor analyses, a “fair” item loading cut-off (0.45) was set (Tabachnick and Fidell, 2007). A 0.2 cut-off was set for communalities (Child, 2006). The Kaiser criterion was used, meaning valid factors should have an eigenvalue greater than one (Costello and Osborne, 2005). For the exploratory factor analysis, examination of the scree-plot and a parallel analysis were also used to corroborate factor solutions. Data from one random split-half of the sample (*n* = 156) was selected for the exploratory factor analysis. Data from the other random split-half of the sample (*n* = 156) was selected for the confirmatory factor analysis. While no clear recommendation exists on what constitutes a satisfactory percentage of explained variance, values between 50 and 60% were chosen to be retained (Peterson, 2000). Goodness-of-fit indices were established prior to testing (Hooper et al., 2008). RMSEA values close to 0.06 were considered indicators of good fit. SRMR values under 0.05 were retained, as well-fitting models tend to obtain similar values. Finally, as it is recommended that a CFI index should be > 0.95, it was decided to keep that value for this testing. Model improvement was assessed through modification index values (MI), implemented in R by the lavaan package. Higher MI values indicate a better fit of the corresponding model, using the LaGrange multiplier.

For inter-item correlations, moderate correlations (0.3–0.7) were considered the lowest acceptable degree of correlation. Internal consistency was evaluated using Cronbach’s alpha and McDonald’s omega. Test–retest fidelity was controlled using absolute agreement intra-class correlations between BISS scores at times 1 and 2, with a two-way mixed model. Convergent validity was determined using Pearson’s correlations between BSQ-8C, EDE-Q, PACS-4 scores and BISS scores. Concurrent validity was assessed using ANOVAs between BMI groups and *t*-tests between eating disorder symptomatology groups. Univariate normality was assessed for *t*-tests. A cut-off interval of [−2; 2] was used as per George and Mallery’s (2010) recommendations. For clinically significant participants, kurtosis was 0.33 and skewness was −0.43. For non-clinically significant participants, kurtosis was −0.61 and skewness was 0.36.

## Results

### Item-Analysis and Construct Validity

**FIGURE 1:**
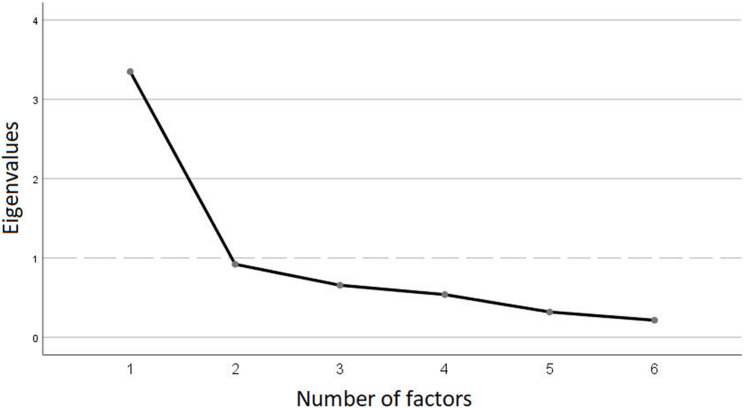
Scree-plot for the F-BISS scale. Dashed line represents the Kaiser criterion.

The exploratory factor analysis was computed using a maximum likeliness extraction method. KMO index was satisfactory (KMO = 0.82), and Bartlett’s test of sphericity produced a significant result (*p* < 0.001). Only one component reached eigenvalue >1.00 (eigenvalue = 3.35) (see Figure 1). A parallel analysis confirmed a one-factor solution (eigenvalue of second factor: 0.92; parallel analysis eigenvalue for a two-factor solution: 1.14). According to the chosen cut-off, item 2 does not adequately load on the main factor (see Table 1). When assessing communalities, item 2 was the only one under our chosen cut-off point (see Table 2). Therefore, item 2 was removed from the rest of the analyses.

**TABLE 1:** Exploratory factor analysis of the F-BISS.

	Factor 1
1. Right now, I feel (Extremely dissatisfied to Extremely satisfied) with my physical appearance	0.90
*En ce moment je me sens (Extrêmement insatisfaite à Extrêmement satisfaite) de mon apparence physique*	
Right now, I feel (Extremely satisfied to Extremely dissatisfied) with my body size and shape	0.26
*En ce moment je me sens (Extrêmement satisfaite à Extrêmement insatisfaite) de la taille et la forme de mon corps*	
Right now, I feel (Extremely dissatisfied to Extremely satisfied) with my weight	0.73
*En ce moment je me sens (Extrêmement insatisfaite à Extrêmement satisfaite) de mon poids*	
Right now, I feel (Extremely physically attractive to Extremely physically unattractive)	0.74
*En ce moment je me sens (Extrêmement physiquement attirante à Extrêmement physiquement repoussante)*	
Right now, I feel (A great deal worse to A great deal better) about my looks than I usually feel	0.65
*En ce moment je me sens (Beaucoup moins bien à Beaucoup mieux) à propos de mon apparence que d’habitude*	
Right now, I feel (A great deal better to A great deal worse) than the average person looks	0.72
*En ce moment je me sens (Beaucoup mieux à Vraiment moins bien) que la moyenne des gens n’en a l’air*	

*Eigenvalue of factor 1 = 3.35.*

*Percentage of variance explained = 56%.*

**TABLE 2:** Communalities for the exploratory factor analysis of the F-BISS.

	Initial	Extraction
Item 1	0.70	0.82
Item 2	0.07	0.07
Item 3	0.53	0.54
Item 4	0.56	0.55
Item 5	0.38	0.42
Item 6	0.53	0.52

**FIGURE 2:**
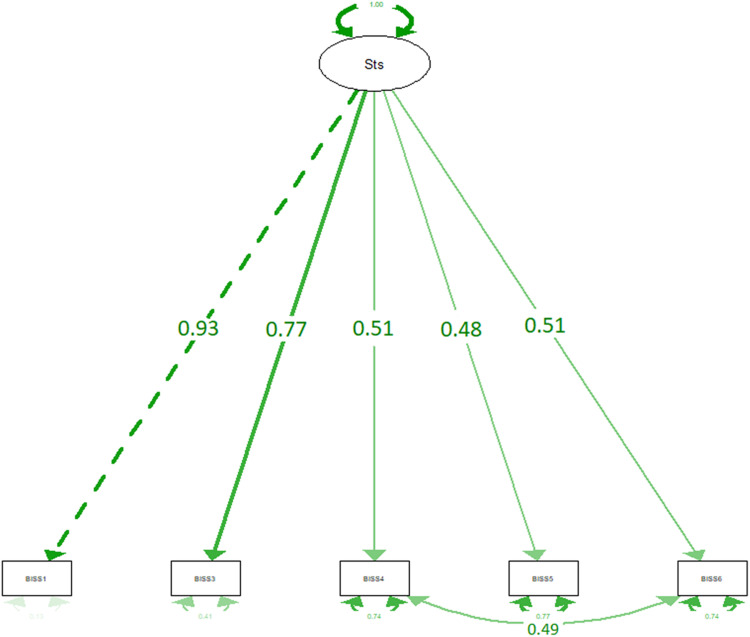
Confirmatory factor analysis of the BISS (model 2). Sts, main factor. One-way arrows represent factor loading. Two-way arrows represent correlations between items.

The confirmatory analysis also returned a single factor solution (see Figure 2). The MI function on RStudio indicated higher MI values when allowing correlation between items 4 and 6 (MI = 37.86). When allowing correlation between items 4 and 6, goodness-offit indices were adequate for the second model (see Table 3). All items had high item-total correlation coefficients at time 1, ranging from *r* = 0.65 (*p* < 0.001) to *r* = 0.85 (*p* < 0.001; see Table 4).

**TABLE 3:** Confirmatory factorial analysis, and goodness-of-fit indices of the F-BISS.

		Factor 1 (model 2)
Item 1		0.93
Item 3		0.77
Item 4		0.51
Item 5		0.48
Item 6		0.51
	
	**Model 1 (without item 2)**	**Model 2 (without item 2; item 4–item 6)**
	
χ^2^(df)	47.14 (5)	7.92 (4)
CFI	0.85	0.99
RMSEA	0.23	0.08
RMSEA CI	[0.18; 0.30]	[0.00; 0.16]
SRMR	0.09	0.03

*–: correlation between items allowed by modification index analysis. CI, confidence Interval.*

**TABLE 4:** French Body Image State Scale items inter-correlations and item-total correlations (ITC).

	Item 1	Item 2	Item 3	Item 4	Item 5	Item 6	ITC
Item 1	–						
Item 2	0.26***	–					
Item 3	0.72***	0.21***	–				
Item 4	0.57***	0.30***	0.39***	–			
Item 5	0.50***	0.15**	0.44***	0.36***	–		
Item 6	0.55***	0.24***	0.41***	0.65***	0.42***	–	
ITC	0.85***	0.53***	0.76***	0.74***	0.65***	0.74***	–

**p < 0.01, ***p < 0.001.

### Reliability

Cronbach’s alpha and McDonald’s omega were used to define the F-BISS’s internal consistency. At time 1 and time 2, internal consistency without item 2 was satisfactory, with α = 0.83 at both time 1 and time 2. Using McDonald’s omega, internal consistency remained satisfactory, with ω = 0.85 at time 1, and ω = 0.86.

The test–retest reliability of the F-BISS was calculated without item 2 over a 2-week period. The coefficient of correlation was *r* = 0.86 (*p* < 0.001) for single measures.

### Convergent Validity

Pearson’s correlations were used to establish links between the F-BISS and BMI, trait measures of body image, measures of social comparison (PACS-4) and measures of eating disorder symptomatology (EDE-Q). F-BISS scores without item 2 strongly and negatively correlated with body dissatisfaction (BSQ; *r* = −0.74, *p* < 0.001), eating disorder symptomatology (EDE-Q; *r* = −0.72, *p* < 0.001), and moderately correlated with BMI (*r* = −0.36, *p* < 0.001), and comparison to others (PACS-4; *r* = −0.43, *p* < 0.001).

Differences between BMI categories were significant (*F* = 13.85; *p* < 0.001), with Bonferroni *post hoc* testing revealing no significant differences between underweight and normal categories (*p* = 0.86), no significant differences between overweight and obese categories (*p* = 1.00), but significant differences between underweight/normal and overweight/obese categories (*p* < 0.001).

Finally, differences between women with clinically significant and non-clinically significant symptomatology for eating disorders were significant (*t* = 11.65; *p* < 0.001). The clinically non-significant group had significantly higher satisfaction.

## Discussion

The results yielded showed the French version of the BISS (F-BISS) to have good psychometric qualities. Items 1, 3, 4, 5, and 6 were adequately loaded on a main factor (>0.45), moderately to strongly intercorrelated, and internal consistency was good after removing item 2. A one-factor solution also showed adequate fit after removal of item 2, and allowing for correlation between items 4 and 6.

The F-BISS was negatively correlated to overall EDE-Q score. Moreover, participants with a clinically significant symptomatology were less satisfied than other participants. This is consistent with eating disorder literature, as a lower F-BISS score indicated lower body satisfaction, and thus greater dissatisfaction, a risk factor in eating disorder development (Stice et al., 2011). As with the Spanish validation of the scale (Mebarak Chams et al., 2019) the F-BISS was negatively correlated to a trait measure of body dissatisfaction (BSQ). Finally, the F-BISS was negatively correlated with social comparison. Again, this is consistent with research (Rodgers et al., 2011) as the original BISS, and thus the F-BISS, measure body satisfaction (Cash et al., 2002). BMI’s correlation to the F-BISS was somewhat consistent with the Spanish validation (*r* = −0.28; Mebarak Chams et al., 2019) but not with the original validation (*r* = −0.53; Cash et al., 2002). Furthermore, an ANOVA showed that significant differences in F-BISS scores between BMI groups lie between “clusters” formed by the underweight and normal groups, and the overweight and obese groups. These findings could indicate either that BMI underestimates obesity prevalence by categorizing obese people as overweight (Shah and Braverman, 2012), or that BMIs in the original study displayed stronger differences between groups, perhaps explained by a possibly higher prevalence of female obesity in the United States than in France (Diouf et al., 2010; An, 2014). In that case, it would be beneficial to assess differences in overall BISS score between American and French samples; even if western countries are supposed to display similar rates of body dissatisfaction (Holmqvist and Frisén, 2010), a higher obesity prevalence could be linked to higher body dissatisfaction in one population over another (Weinberger et al., 2016). Finally, results showing a significant difference between participants with a clinically significant eating disorders symptomatology score are coherent with research establishing body dissatisfaction as a risk-factor of eating disorder development (Stice, 2002; Stice et al., 2011).

Test–retest reliability was also slightly higher than the original scale with a 2-week interval (*r* = 0.69; Cash et al., 2002), and comparable to the first test–retest coefficient from the Italian validation with an hour interval (*r* = 0.87; Carraro et al., 2010). This may imply that this translation is more stable than expected. In both our study and the original study, test–retest reliability was assessed in a neutral “questionnaire-filling” context. However, in our study, participants had to fill the questionnaire at home using a computer, while they had to come to a laboratory in the original study. Perhaps the presence of the research team in the original study could have induced social comparison of appearance before the experiment, causing a bias as social comparison of appearance is linked to lesser body satisfaction (Myers and Crowther, 2009). State-mood, a variable we have not controlled, has been shown to have an effect on state body dissatisfaction; when an individual feels better, their body dissatisfaction tends to be lower (Colautti et al., 2011). With this in mind, it could be more stable in a familiar environment like the one in our study than in a laboratory context. Moreover, we have not verified if scores would remain stable after subjecting participants to different contexts, like in the original study (Cash et al., 2002). Finally, perhaps testing the scale with shorter retest intervals could be appropriate, to reduce eventual lability (Polit, 2014).

The research conducted presents some limitations. Firstly, it has not been tested in a male population, unlike every other validation study. This poses a concern with regard to generalization. As mentioned, the reason for men’s exclusion is that a meta-analysis has shown that men present higher dissatisfaction about muscularity (Karazsia et al., 2017). This study would have required other measures of convergent validity for men, as the scales used were focused on thin appearance and weight loss, and were validated in a female population (Dany and Urdapilleta, 2012; Lentillon-Kaestner et al., 2014; Carrard et al., 2015). Secondly, the research has never been tested within a broader age group. This should be corrected in future studies by using appropriate scales. Indeed, older women are also affected by body-image issues (Marshall et al., 2012), despite being less thinness-driven than younger women (Pruis and Janowsky, 2010). Thirdly, contrary to the Spanish translation (Mebarak Chams et al., 2019), item 2 had to be deleted due to insufficient factor loading and low inter-item correlations. This item made reference to body size and shape. It was surmised after the study that “body size and shape” should have simply been translated as “*silhouette*” and not “*taille et forme du corps.*” The latter translation was thought to be more literal, and closer to the original English phrasing. However, this item’s removal does not seem detrimental to the overall validity of the scale, as borne out in section “Results,” making corrections probably unnecessary. Finally, EDE-Q cut-off scores used in this study were validated in a Norwegian adult sample, not a French sample. While results are coherent with literature, it would be needed to establish psychometric norms in a French population to provide more reliable cut-off scores. Moreover, a clinically significant EDE-Q score cannot be considered an eating disorder diagnosis. Therefore, it would be needed in future research to assess F-BISS scores in women with and without clinically established eating disorders.

Future studies should focus on replicating the original study’s protocol, and test the F-BISS in different situational contexts, such as a day on the beach, or reading a fashion magazine alone. Indeed, the F-BISS was only tested in a neutral (questionnaire filling) situation without any physical intervention of the research team, which could explain its relative stability. When the situational context is positive for body image, F-BISS scores should be higher, indicating body satisfaction (Cash et al., 2002). When the situational context is negative or threatening for body image, F-BISS scores should be lower. Other measures of convergent validity should also be used, such as indicators of thin-ideal internalization. The SATAQ-4R (Schaefer et al., 2017) or the DKB-35 (Zohar et al., 2017; Lev-Ari et al., 2020), another trait-measure of body image, would be adequate choices. Another measure of convergent validity that should be used is state-mood. As said earlier, state-mood is positively linked to state body-dissatisfaction (Colautti et al., 2011). Conducting further analyses in other ethnic groups, age groups, genders, or other French-speaking samples, such as Belgian or French-Canadian samples, could prove worthwhile for generalization. Finally, validation in an eating disorder diagnosed population and comparison with a general population would allow verification of our *t*-test results in a clinical setting.

In a research setting, this scale could be used to identify, for instance, the effect of exposure to body image-threatening content in 18–25-year-old females. In a clinical setting, such as eating disorder treatment, its ability to measure state body image satisfaction would allow for enhanced monitoring of the patient’s state, and a more comprehensive understanding of the daily life situations that increase or decrease body satisfaction.

## Data Availability Statement

The raw data supporting the conclusions of this article will be made available by the authors, without undue reservation.

## Ethics Statement

The studies involving human participants were reviewed and approved by the Université de Lille ethical review board (ref: 2019-345-S71). The patients/participants provided their written informed consent to participate in this study.

## Author Contributions

LB: original draft, methodology, recruitment, conceptualization, and reviewing and editing. CA: reviewing and editing, recruitment, methodology, and conceptualization. CB and FL: reviewing and editing and formal analysis. AR: data supervision and curation, project administration, reviewing and editing, and formal analysis. All authors contributed to the article and approved the submitted version.

## Conflict of Interest

The authors declare that the research was conducted in the absence of any commercial or financial relationships that could be construed as a potential conflict of interest.

## Publisher’s Note

All claims expressed in this article are solely those of the authors and do not necessarily represent those of their affiliated organizations, or those of the publisher, the editors and the reviewers. Any product that may be evaluated in this article, or claim that may be made by its manufacturer, is not guaranteed or endorsed by the publisher.
